# Foliar calcium application reduces fluorine accumulation in tea plant by regulating cell wall structure and gene expression

**DOI:** 10.3389/fpls.2024.1443439

**Published:** 2025-01-03

**Authors:** Jinlei Luo, Lintao Zhang, Daili Li, Shuangjie Huang, Chunlei Li, Yi Chen, Peng Yin, Wei Liu, Mufang Sun, Guiyi Guo

**Affiliations:** ^1^ Xinyang Agriculture and Forestry University, Henan Key Laboratory of Tea Plant Comprehensive Utilization in South Henan, Xinyang, Henan, China; ^2^ Dabie Mountain Laboratory, Xinyang, Henan, China; ^3^ Shandong Provincial University Laboratory for Protected Horticulture, Weifang University of Science and Technology, Shouguang, Shandong, China; ^4^ Xinyang Wenxin Tea Co., Ltd., Xinyang, Henan, China

**Keywords:** tea plant, fluorine, calcium, cell wall, transcriptomics

## Abstract

Tea plant can enrich a large amount of fluorine (F) in the cell wall of its mature leaves, thus posing the risk of excessive intake of F for tea consumers. This study investigated the effect of foliar calcium (Ca) application (0.05-1 mM) on F accumulation in tea plant leaves by analyzing the association of F with cell wall materials, pectin methylesterification structure, and cell wall genes. Ca spray could effectively reduce the F level, the content of wall carbohydrates (excluding the chelate pectin fraction) and the pectin methylesterification degree in tea plant leaves. Correlation analysis further revealed that the tea leaf F content was positively correlated with most of the cell wall materials. Transcriptomics analysis indicated that the key genes involved in reorganization of cell wall polysaccharides (such as *CslE6*, *XTH32*, *PG*, *PGIP*, *PME18*, *PMEU1*, and *PMEI9*) were associated with the variations in F content and cell wall components. All the results suggest that Ca may lessen the tea leaf F level by reducing the accumulation of cell wall materials and intervening the cell wall structure and gene expression. This study provided useful information for reducing the F level and solving the F safety problem in tea plant.

## 1 Introduction

Tea, the most popular and widely consumed beverage worldwide, is favored for its unique flavor and excellent health functions. However, tea plant has the characteristic of high fluorine (F) enrichment in its mature leaves, and F is a nonmetallic element with some health benefits when ingested at a small amount, but its excess intake can cause diseases like dental fluorosis and skeletal fluorosis (Ozsvath, 2009). The tea consumers may intake excessive F from some tea products processed from mature tea leaves (such as brick tea) and run the risk of endangering their health, suggesting the importance to find effective measures to reduce the tea leaf F level.

In recent years, some researchers have found that calcium (Ca) application can significantly reduce F accumulation in tea plant, but the mechanism associated with the regulatory effect of Ca on F remains poorly understood (Ruan et al., 2004; Zhang et al., 2016; Luo et al., 2019). Ca is an essential macronutrient required for cell wall and membrane structure, growth and development regulation, and stress responses (Hepler, 2005). Previous studies have suggested that Ca applications could induce plant tolerance to abiotic stress, such as extreme temperature stress (Shi et al., 2014; Li et al., 2015) and ion toxicity stress (Patel et al., 2011; Vaghela et al., 2009, 2010). Under Ca intervention, the influenced cell wall properties are known to be one of the most important factors for the enhanced adaptation of plants to stressful growth conditions (Porto et al., 2013). The plant cell wall is mainly composed of three types of polysaccharides (cellulose, hemicellulose and pectin) and the pectin can be linked by Ca in a demethylesterified form under catalysis of pectin methylesterase (PME) to form higher structures, exerting an important effect on wall integrity and characteristics such as porosity, rigidity and adhesion (Min et al., 2016). Several studies have indicated that exogenous Ca application can influence the morphology and function of plant cells by regulating the cell wall composition, structure and related genes expression (e.g. *PMEs*) (Martins et al., 2020; Hou et al., 2022). Interestingly, researches have established that the cell wall is the main F binding site in tea plant leaves, with 71%-92% of tea leaf F distributed in the cell wall (Hu et al., 2019; Luo et al., 2021). Moreover, Luo et al. (2020, 2021) treated tea plants with different concentrations of F and found that the composition and structure of cell wall polysaccharides, cell wall related metabolism and gene expression were greatly influenced by F and showed significant correlations with the F content in tea leaves. Based on these reports, the reducing effect of Ca on F level in tea plant can be speculated to be linked to variations in cell wall structure and composition. However, to our best knowledge, no study has ever been performed on the effect of Ca on tea plant cell wall or the relationship between F content regulation by Ca and cell wall structure.

Therefore, this study aimed to reveal the association of varied F levels with cell wall components, structure and gene expressions by foliar Ca spray at different concentrations, to explore the mechanism of Ca towards reducing the tea leaf fluorine and to provide effective reference for its practical application in tea plant defluorination.

## 2 Materials and methods

### 2.1 Plant materials and calcium supplements

The experiments were carried out with 2-year-old “Fudingdabaicha”, a popular tea variety originating in Fujian province and widely cultivated in China. The tea plants were hydroponically cultivated with nutrient solution using a formula without containing Ca reported by Luo et al. (2021). After adaptation of two weeks, both leaf surfaces of tea seedlings were sprayed evenly three times a day for two weeks with purified water (control) and 0.05, 0.25, 0.5, and 1 mM Ca(NO_3_)_2_ (defined as CK, Ca0.05, Ca0.25, Ca0.5, and Ca1, respectively). In addition, NH_4_NO_3_ (1, 0.95, 0.75, 0.5, and 0 mM) was used to ensure the same nitrogen supply in each Ca treatment. Next, tea seedlings in different Ca treatments were cultured for 24 h with nutrient solution containing 0.4 mM NH_4_F, followed by collecting the leaves for subsequent analysis. In current study, the chemical Ca(NO_3_)_2_ and NH_4_F were chosen as the Ca and F source respectively on the basis of their wide use in similar experiments (Yang et al., 2015; Luo et al., 2019; Huai et al., 2022). Moreover, we selected the F supply concentration at 0.4 mM to ensure sufficient source of F in nutrient solution and not causing harm to the tea plant simultaneously according to the research of Yang et al. (2015).

### 2.2 Physiological index detection

The malondialdehyde (MDA) content was detected using the thiobarbituric acid (TBA) method as described by Dhindsa et al. (1981). The activity of the superoxide dismutase (SOD) enzyme was detected using the corresponding detection kits according to the manufacturer’s instructions (Nanjing Jiancheng Bioengineering institute, China).

### 2.3 Determination of fluorine and calcium content in tea leaves

To determine F and Ca content, fresh leaf samples were fixed at 105 ℃ for 10 min, followed by thoroughly drying at 60 ℃ by a dryer and then grinding into a powder. Finally, the F ion was leached from the tea powder using a previously reported alkali ashing method and quantified using a fluoride ion selective electrode (F090, Thermo, US). The Ca content were determined using an inductively coupled plasma emission spectrometer (ICP6300, USA) after dry-ashing the powder and diluting it to a standard volume with deionized water (Luo et al., 2019). Values were normalized on a dry weight basis.

### 2.4 Cell wall isolation and polysaccharides content detection

The extraction of cell wall and isolation of polysaccharides from cell wall were conducted as previously reported (Luo et al., 2020). Briefly, cell walls in different tea leaf samples were extracted as an alcohol insoluble solid (AIS) by homogenizing fresh leaves with alcohol and washing successively with acetone, methanol: chloroform (1:1, v/v) and methanol. Next, the extracted cell wall materials were freeze-dried and weighed, followed by sequential extraction with 50 mM imidazole, 50 mM Na_2_CO_3_ and 4 M KOH to obtain chelate pectin, alkali-soluble pectin and hemicellulose, and the residue was made into a suspension with pure water and defined as cellulose. The amount of galacturonic acid (GalA) in chelate pectin and alkali-soluble pectin fractions was determined to express the content of each pectin component, and the amount of neutral sugar in hemicellulose and cellulose fractions was detected to estimate the content of the two polysaccharides. The GalA and neutral sugar were assayed by the m-hydroxybiphenyl method and the anthrone method using GalA and glucose as the standard, respectively (Blumenkrantz and Asboe-Hansen, 1973; Yemm and Willis, 1954).

### 2.5 Determination of monosaccharides in cell wall of tea leaves

The cell wall monosaccharides, including galacturonic acid, glucose, galactose, arabinose, xylose, mannose, rhamnose and fucose, were determined by liquid chromatography using their pure materials as standards. Specifically, 50 mg cell wall was placed in a hydrolysis tube and acidified with 2.0 mL 78% sulfuric acid for 1 h, followed by dilution in water to 50 mL, filling with nitrogen, and hydrolysis in an oven at 110 ℃ for 12 h. Next, 0.5 mL cooled hydrolysate was added to a 4 mL centrifuge tube, adjusted to neutral pH with NaOH and set to 1 mL. After adding 0.2 mL 0.3 M NaOH and 0.4 mL PMP methanol solution, the tube was filled with nitrogen and then immersed in a 70 ℃ water bath for 60 min and then cooled to room temperature. Finally, 0.2 mL 0.3 mol/L HCl was added to the solution and the volume was fixed to 2 mL, followed by three washes with chloroform and passing the water layer through a 0.45 μm filter membrane. Analysis was performed with high performance liquid chromatography (HPLC) (Agilent 1200, Agilent, US) using a TC-C18 column (4.6 mm x 250 mm x 5 mm, Agilent, USA). The mobile phase A was 0.1 mol/L KH_2_PO_4_ (pH6.8) and the mobile phase B was acetonitrile. The samples were isocraticly eluted with a mobile phase gradient of A:B=82:18, at a flow rate of 1.0 mL/min, a column temperature of 25 ℃, an injection volume of 10 μL, and a detection wavelength of 254 nm.

### 2.6 Pectin methylesterification structure analysis

To observe the pectin methylesterification structure, immunohistochemical experiments were performed for locating and evaluating the signal of low- and high-methylesterified pectin by using monoclonal antibodies LM19 and LM20, respectively. Briefly, tea leaf tissue sections were fixed by 4% paraformaldehyde, embedded in paraffin and sectioned at 8 μm thickness, followed by dewaxing and rehydration. Then, the segments were treated successively with 3% nonfat dry milk in 0.01 M phosphate-buffered saline (PBS) for 1 h, the primary (LM19 and LM20) and secondary (goat anti-rat) antibodies for 2 h, and DAPI staining solution for 5 min at room temperature, with an extensive 0.01 mol/L PBS wash after each treatment. Next, the sections were observed with a fluorescence microscope (Axio Observer 3 V20, Zeiss, Germany) with narrow band filtering for AlexaFluor 488 and DAPI. The primary antibodies were purchased from Kerafast (Beijing, China) and the secondary antibody were purchased from Jackson (Pennsylvania, USA), both of which were used as instructed by the manufacturers. Negative controls were conducted by omitting the primary antibodies, and non-specific binding of LM19 and LM20 was observed. All the images were captured at the same magnification, and the fluorescence intensity of LM19 or LM20 was determined using Image J (NIH, US). The experiments were repeated three times to ensure the repeatability of the results.

The degree of methylesterification (DM) of pectins (i.e. the percentage of methylesterified galacturonic acids to the total galacturonic acids) was assessed by quantification of the released methanol according to the method of Fernandes et al. (2013). Specifically, 20 mg cell wall was dissolved in 1.5 ml distilled water and incubated by 0.5 ml 1.5 M NaOH at room temperature for 30 min, followed by chilling on ice and adding with 0.5 ml 4.5 M H_2_SO_4_. Next, 0.5 ml supernatant of the samples after centrifugation was mixed in sequence with 0.5 ml 0.5 M H_2_SO_4_ and 0.2 ml 2% KMnO_4_ (w/v) in an ice bath environment and left for 15 min. The samples were added with 0.2 ml 0.5 M sodium arsenite in 0.06 M H_2_SO_4_, mixed and left for 1h at room temperature, added with 2ml acetylacetone-ammonium acetate in tubes and the tubes were vortexed, closed and heated at 59 ℃for 15 min. Finally, the samples were cooled to room temperature and the absorbance was recorded at 412 nm to determine the methanol content. An equal amount of cell wall was used to determine the content of total galacturonic acids and the DM was calculated as the ratio of the moles of methanol to galacturonic acid.

### 2.7 Pectin methylesterase activity determination

The PME activity was determined according to Yang et al. (2019) with some modifications. Briefly, tea leaf samples were ground in ice with 5 mM phosphate buffer containing 1 M NaCl and 5% PVP (pH 7.5) and incubated on ice for 1 h with repeated vortexing and then centrifuged at 4 ℃ to collect the supernatent. The protain concentrations in the supernatent were detected using a BCA Protein Concentration Determination Kit. Next, 100 μL supernatant was mixed with 3.90 mL PME activity assay buffer containing 0.5% citrus pectin, 0.2 M NaCl, and 0.015% methylred (pH 6.8), followed by measurement of the absorbance at 525 nm before and after incubation at 37 ℃ for 1.5 h. A calibration curve was obtained by adding 10-300 μL 0.01 M HCl to the 4 mL PME activity assay buffer. The PME activity was expressed as the amount of released hydrogen ions per time unit and per protein concentration (in mg).

### 2.8 Transcriptome analysis

For transcriptomics analysis, total RNA was extracted from leaf samples of different Ca treatments using the Quick RNA Isolation Kit (Huayueyang Biotechnology Co., Ltd., Beijing, China) with three replicates. The mRNA was gathered from the total RNA by Oligo (dT) magnetic bead enrichment, randomly interrupted with divalent cations in the NEB fragmentation buffer and sent for library construction. The cDNA libraries were constructed using the Agilent 2100 bioanalyzer and sequenced on an Illumina HiSeq TM2000 platform. Next, the clean reads were obtained by filtering the original sequencing data and checking the sequencing error rate and GC content distribution, followed by aligning to the reference genome using the HISAT2 software (Wei et al., 2018). The gene expression value of RNA-seq was calculated based on the number of fragments per kilobase of transcript sequence per million base pairs (FPKM) and the differentially expressed genes (DEGs) between Ca spraying treatments and the control without Ca supply were identified using DESeq with a standard of |log2 FC (fold change) | > 1 and p < 0.05. The Venn diagrams were drawn online (https://magic.novogene.com) to screen for the key overlapping DEGs to compare the Ca treatments with the control. Additionally, Kyoto Encyclopedia of Genes and Genomes (KEGG) enrichment analyses of the key DEGs were performed by using the clusterProfiler software to find the important metabolic pathways.

### 2.9 Quantitative real-time polymerase chain reaction analysis

To verify the reliability of RNA-Seq experiments, the relative expression of five cell wall-related genes was detected by qRT-PCR. Briefly, total RNA (1 μg) in each fresh sample was reverse transcribed to cDNA by using a TRUEscript 1st Strand cDNA Synthesis kit (Aidlab, BeiJin). Then, the qRT-PCR was carried out using a Sybr Green qPCR Mix (Aidlab, BeiJin) on a StepOnePlusTM Real-Time PCR instrument (StepOnePlus, Singapore) as recommended by the supplier. PCR amplification was carried out with 40 cycles of 95 ℃ for 10 s (denaturation), 63 ℃ for 30 s (annealing), and 72 ℃ for 30 s (extension). The glyceraldehyde 3-phosphate dehydrogenase (GAPDH) was used as an internal control due to its high stability in our experimental materials. The relative values of gene expression of each mRNA were calculated by normalization to the GAPDH expression using the 2^–ΔΔCT^ method and expressed as fold values of the control. Forward and reverse primer sequences are shown in **Supplementary Table S1**.

### 2.10 Statistical analysis

The differences between various treatments were assessed using a one-way analysis of variance (ANOVA) followed by Duncan’s test using the SPSS 19.0 software. The correlation among the F level, cell wall component content, and gene expression was analyzed using the SPSS 19.0 software. The Pearson correlation coefficients obtained from this analysis were used for constructing the heat map. Backward stepwise multiple linear regression (MLR) was conducted for developing an F accumulation model based on the cell wall components and genes expression (Hernandez-Hierro et al., 2014).

## 3 Results

### 3.1 Effect of calcium spray on the MDA content and SOD activity of tea plant leaves

**Figure 1:**
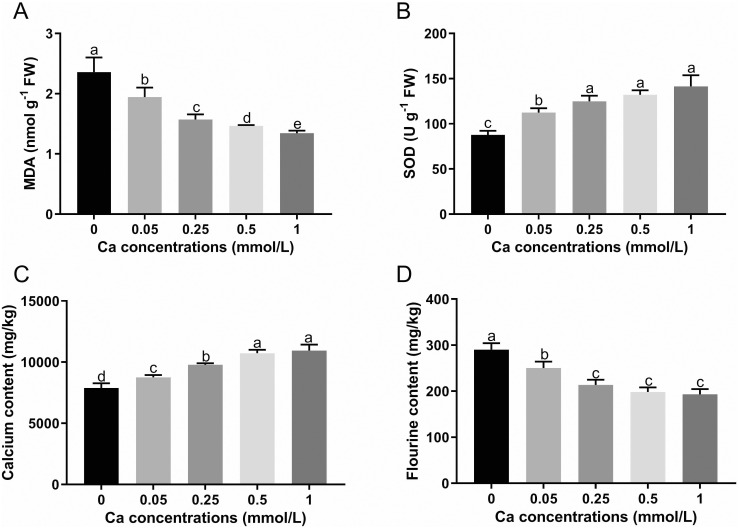
The MDA content **(A)**, SOD activity **(B)** and the content of calcium **(C)** and fluorine **(D)** in tea leaves under different calcium concentrations. Data are shown as means ± SD of three replicates. Different letters above the column indicate significant difference (p<0.05) between treatments.

The MDA content in tea plant leaves was significantly decreased as the concentration of the foliar calcium application was increasing (**Figure 1A**). On the contratry, the SOD activity was remarkably increased with calcium concentration, although the change was not significant in the range of 0.25 to 1 mM Ca treatments (**Figure 1B**).

### 3.2 Effect of calcium spray on fluorine and calcium content in tea plant leaves

With increasing exogenous Ca concentration, the Ca content showed an increasing trend in the leaves of tea seedlings, with an increase of 11.00%, 24.15%, 36.12% and 38.86% in the Ca present treatments (0.05, 0.25, 0.5, and 1 mM Ca) respectively versus the control (0 mM Ca), indicating that the Ca content in the leaves of tea plant is significantly affected by the Ca spray level (**Figure 1C**). As shown in **Figure 1D**, Ca spray could significantly reduce the F content in tea plant leaves with a reduction rate of 17.24% to 33.33% versus the control. The F content in tea leaves was gradually decreased with increasing Ca concentration, despite no significant changes in the Ca range of 0.25 to 1. The results showed that foliar Ca application can increase the Ca level and lower the F level in tea plant.

### 3.3 Effect of calcium spray on content of cell wall polysaccharides and monosaccharides in tea plant leaves

**Figure 2:**
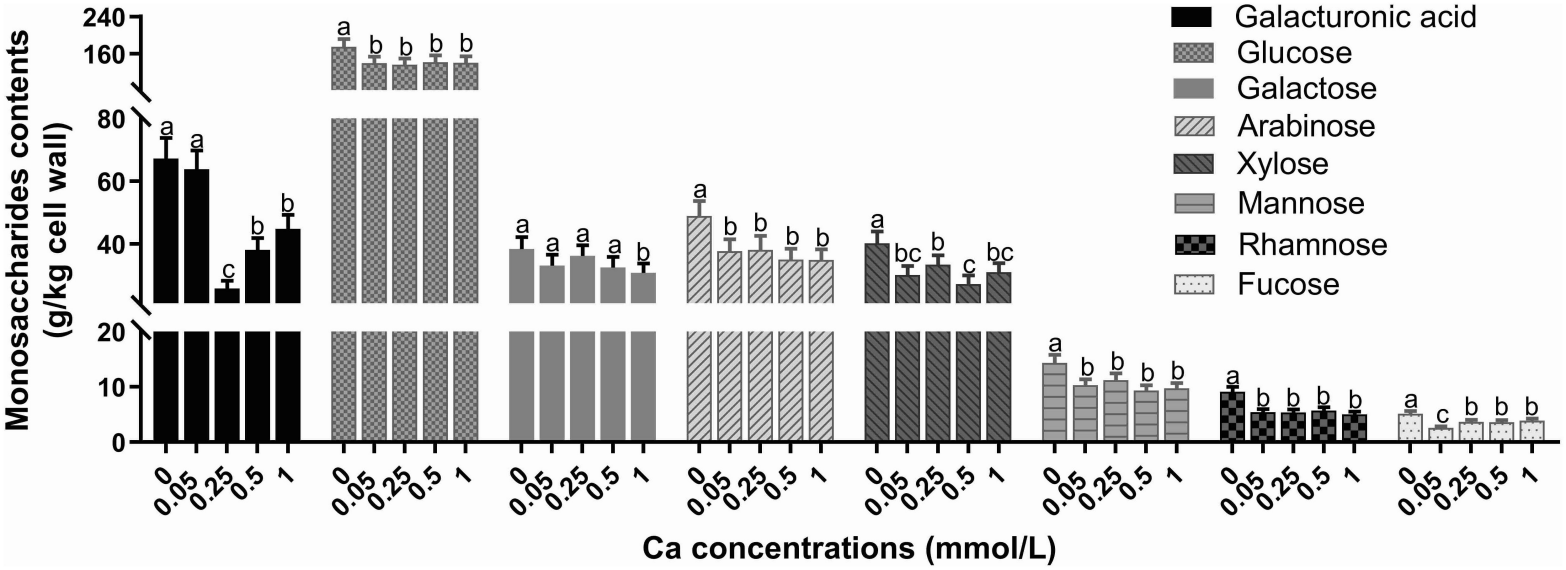
Effect of different calcium treatments on the content of cell wall monosaccharides. Different letters above the column indicate significant difference (p<0.05) between treatments.

As shown in **Table 1**, the contents of both tea leaf cell wall and cellulose were diminished by spraying Ca at any concentration. With increasing Ca concentration, the cell wall content followed the trend of decreasing first and then increasing, reaching the minimum at 0.25 mM. In terms of polysaccharides, cellulose was gradually reduced, and both alkali-soluble pectin and hemicellulose showed similar changes, with a lower content in the 0.05-0.5 mM Ca concentration range, but a significant enhancement (*p<*0.05) at 1 mM Ca concentration versus the control. Note that the chelate pectin was remarkably promoted by Ca spray and its changing law with Ca was exactly contrary to that of the cell wall. Collectively, Ca supply had a depression effect on the quantity of all the cell wall monosaccharides (**Figure 2**). Specifically, the content of galacturonic acid fell first and then rose, reaching the minimum value at 0.25 mM. The content of glucose, arabinose, mannose and rhamnose was remarkably lower in the Ca-present treatments versus the control, but with no significant difference among the Ca-treated groups. The galactose content showed only a significant decrease at 1 mM Ca concentration. The change of xylose showed no regularity, with lower values at Ca concentrations of 0.05 and 0.5 mM. Additionally, with increasing Ca, the fucose content showed a decrease first, followed by an increase, with the minimum level at 0.05 mM Ca. The above results reflected that Ca had an overall inhibitory effect on the accumulation of cell wall materials (i.e., AIS, cellulose, monosaccharides) while a promoting effect on the chelate pectin component.

**Table 1:** Content changes of cell wall and its polysaccharide components in tea plant leaves treated by different concentrations of calcium.

Calcium (mM)	Cell wall (mg/g FW)	Chelate pectin (mg/g CW)	Alkali-soluble pectin (mg/g CW)	Hemicellulose (mg/g CW)	Cellulose (mg/g CW)
0	252.96 ± 10.12 a	3.85 ± 1.16 d	19.60 ± 0.96 b	32.37 ± 1.51 b	132.93 ± 7.59 a
0.05	211.56 ± 9.86 b	8.63 ± 0.19 a	12.18 ± 0.48 c	29.28 ± 1.55 c	118.13 ± 10.51 ab
0.25	204.80 ± 12.33 b	9.81 ± 1.43 a	10.69 ± 0.64 d	30.64 ± 0.95 bc	119.26 ± 14.11 ab
0.5	210.28 ± 8.97 b	7.89 ± 0.07 b	12.58 ± 0.93 c	30.54 ± 3.22 bc	109.10 ± 7.38 b
1	217.04 ± 14.29 b	6.61 ± 0.51 c	25.84 ± 1.21 a	35.92 ± 0.80 a	101.57 ± 6.54 b

Data are expressed as means ± SD of three biological replicates. Different small letters indicate significant difference (p < 0.05) in the treatments versus the control (0 mM Calcium).

### 3.4 Effect of calcium spray on pectin methylesterification structure in tea plant leaves

**Figure 3:**
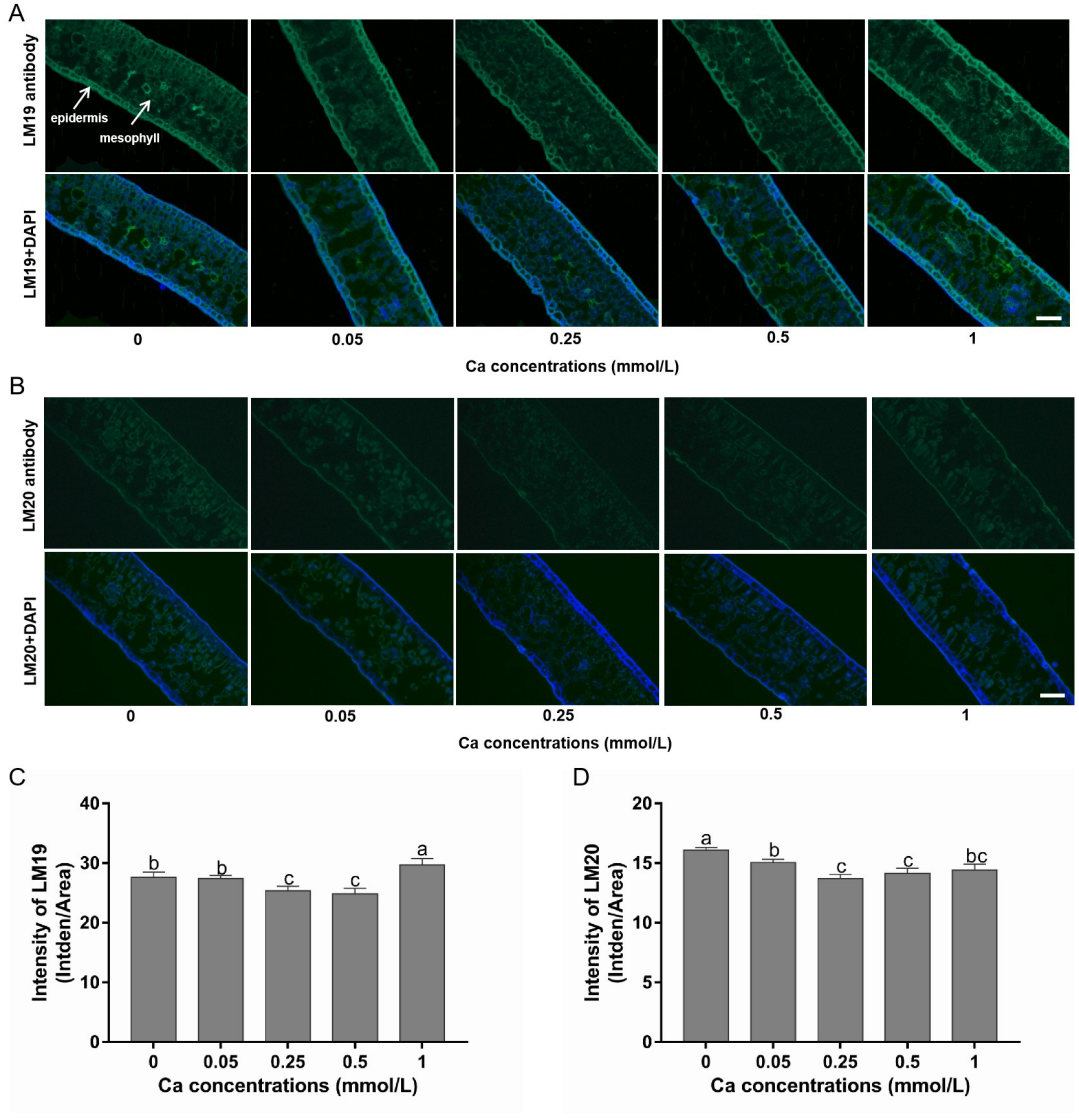
Immunohistochemical staining of low- and high-methylesterified pectin in tea leaves under different calcium conditions. **(A)** Images of the LM19-labeled low-methylesterified pectin alone (in the first row) and in combination with DAPI staining (in the second row) in samples treated with calcium at 0-1 mM; white arrows indicate different leaf tissues. **(B)** Images of the LM20-labeled high-methylesterified pectin alone (in the first row) and in combination with DAPI staining (in the second row) in samples treated with calcium at 0-1 mM. All pictures were captured at the same magnification and the scale bar = 100 μm (shown in the right bottom corner). **(C, D)** Changes in the fluorescence intensity of LM19 **(C)** and LM20 **(D)** under different calcium conditions. Data are expressed as means ± SD of three biological replicates. Different small letters above the bars indicate significant difference (p<0.05) among different treatments.

Pictures of tea tissues from different samples labeled by LM19 and LM20 are shown in **Figures 3A, B**, and the corresponding fluorescence intensities are shown in **Figures 3C, D**. In general, the LM19-labeled low-methylesterified pectin exhibited obviously higher fluorescence signal intensity than the LM20-labeled high-methylesterified pectin, demonstrating that the pectins in tea leaves were mainly present in a demethylated state. Moreover, the distribution differed in the location of the two structures in tea leaf tissues, with more low-methylesterified pectin in the epidermal tissue than in the mesophyll tissue while an even distribution of high-methylesterified pectin across the whole leaf as indicated by the signal intensity of LM19 and LM20 epitopes.

Compared with the control without Ca supply, the fluorescence intensity of LM19-labeled pectin was not affected by 0.05 mM Ca spray, but was significantly reduced by 0.25 and 0.5 mM Ca and increased by 1 mM Ca treatment. This indicated that the formation of low-methylesterified pectin was inhibited by low but promoted by high dose of exogenous Ca (**Figures 3A, C**). Additionally, the intensity of LM20-labeled pectin was significantly lower in all the Ca-present treatments versus the control, following a pattern of decreasing first and then increasing with Ca concentration, reaching the minimum value at 0.25 mM (**Figures 3B, D**). This suggested that Ca had a non-dose-dependent inhibition effect on high-methylesterified pectin.

**Figure 4:**
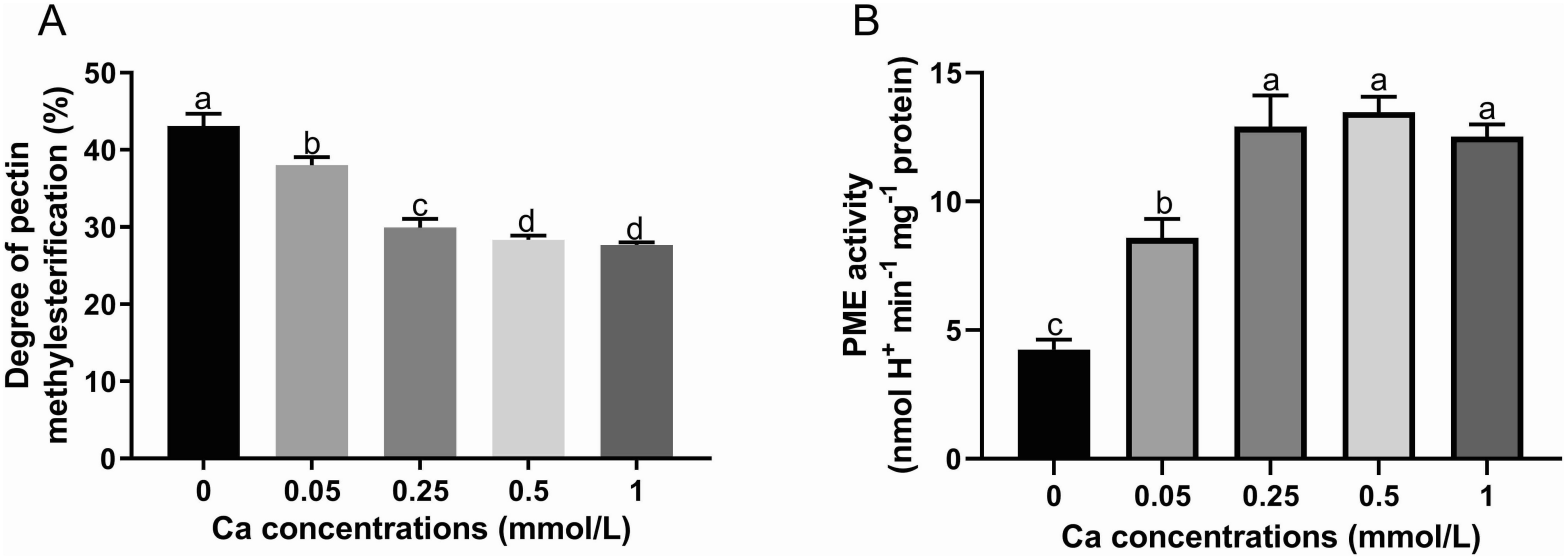
Effect of different calcium treatments on the degree of pectin methyl esterification **(A)** and PME activity **(B)**. Different letters above the column indicate significant difference (p<0.05) between treatments.

The degree of pectin methylesterification (DM) is the ratio between the amount of esterified galacturonic acids and total galacturonic acids in pectin. The DM value decreased significantly with increasing Ca concentration compared to the control without Ca addition and the decreasing trend became slow as the Ca concentration exceeded 0.25 mM and the difference was not significant between Ca concentrations of 0.5 and 1 mM (**Figure 4A**). PME is a key enzyme in the demethylesterification of pectin, its activity was remarkably higher in Ca present treatments compared with the Ca-absent control (**Figure 4B**). The PME activity rose sharply with Ca concentration increasing from 0 to 0.25 mM while was not obviously changed when Ca concentration was at the range of 0.25 to 1 mM. These results suggested that foliar Ca application activated the PME and promoted the de-methylesterified process of pectin.

### 3.5 Effect of calcium spray on transcription level in tea plant leaves

A total of 130.97 Gb clean data (6 Gb for each sample) were obtained, with the percentage of Q30 base above 92% (**Supplementary Table S2**). The clean data were mapped to the reference sequences of tea plant genome (Wei et al., 2018) with an 87% total mapping rate, 82% exon region, 6% intron region, and 13% intergenic region. According to the comparison results and gene location information on the reference genome, the number of reads for each gene was counted and expressed as FPKM. A total of 62,095 transcripts were found to be expressed in the 15 samples, with 41,163 genes mapped to the tea plant genome and 20,932 novel genes predicted by Cufflinks software.

Comparisons of the DEGs between different Ca treatments and the control group without Ca supply are shown in **Supplementary Figures S1A–D**. The number of DEGs was close for Ca-0.25 and Ca-0.5 versus the control (2413 and 2318) and higher than the number between Ca-0.05 and control (2197), with the highest number of DEGs for Ca-1 versus the control (2873). Moreover, the number of down-regulated genes was greater than that of up-regulated genes in the Ca concentration range from 0.05 to 0.5 mM versus the control, but it was just the opposite in the case of Ca-1 versus the control. These results demonstrated that Ca spray has a great effect on the gene transcription of tea plant, and the influence was much higher in the treatments at a higher Ca concentration. All the four combinations shared 744 DEGs according to the Venn diagram (**Supplementary Figure S1E**) and these overlapping DEGs may play an important role in Ca response.

**Supplementary Figure S2** shows the top 20 significantly enriched KEGG pathways in the comparisons of Ca-0.05 (a), Ca-0.25 (b), Ca-0.5 (c) and Ca-1 (d) with the control. “Plant pathogen interaction”, “MAPK signaling pathway” and “plant hormone signal transduction” were the top three metabolic pathways in all the comparison combinations. Moreover, many of the pathways on the list are associated with biosynthesis of carbohydrates and cell wall polysaccharides, including “N-glycan biosynthesis”, “starch and sucrose metabolism”, “galactose metabolism”, “amino sugar and nucleotide sugar metabolism” and “pentose and glucuronate interconversions”. This suggested that the distinguished gene expression patterns under Ca treatments could lead to changes in the structure and content of cell wall components.

**Figure 5:**
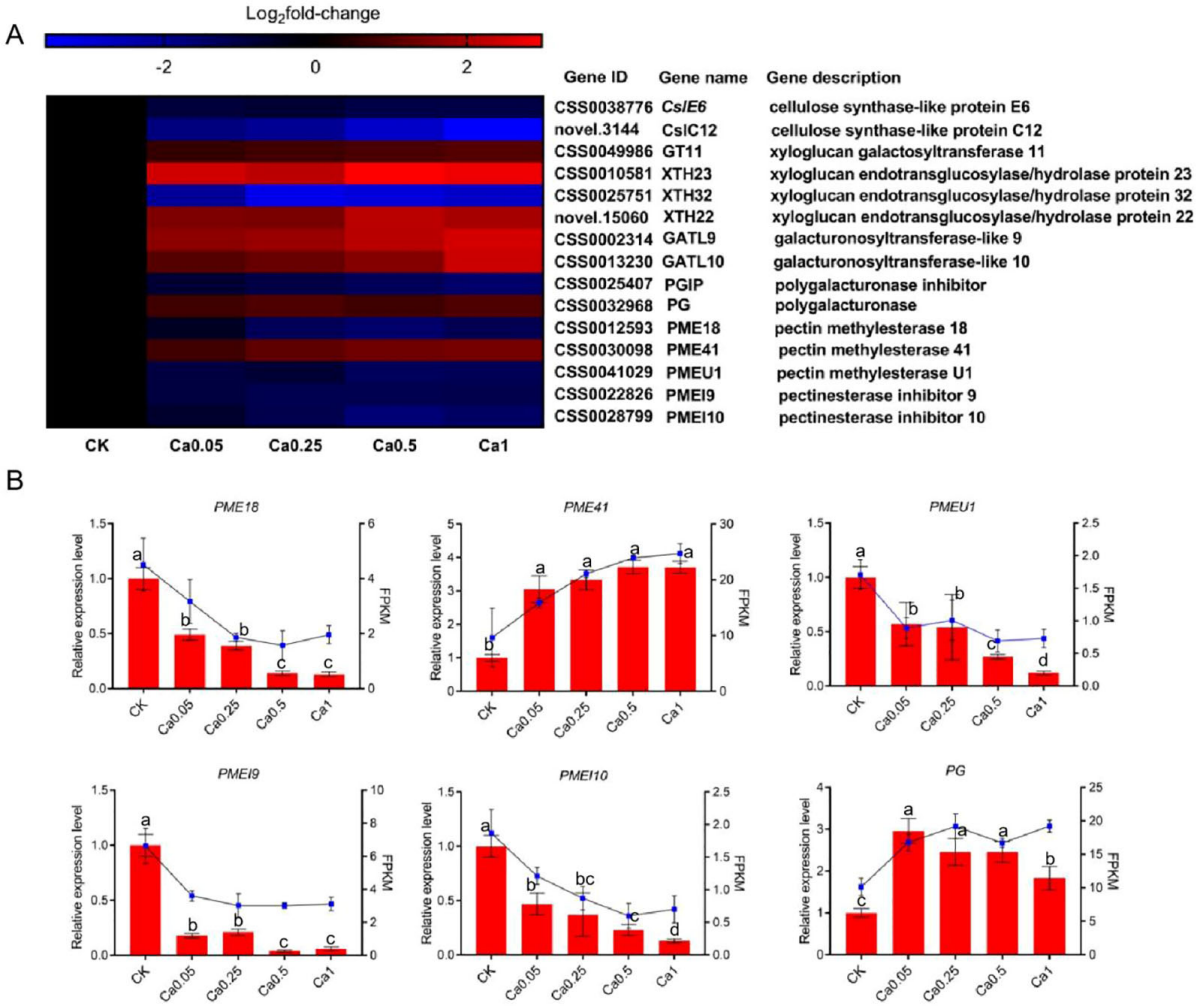
Effect of different calcium treatments on the transcription level of genes involved in cell wall metabolism. **(A)** Heat map of DEG expression profiling from the RNA-Seq data. **(B)** qRT-PCR analysis of DEG expression levels. Data are expressed as means ± SD of three biological replicates. Different small letters above the bars indicate significant difference (p<0.05) among different treatments.

The key DEGs associated with the modification and disassembly of cell wall polysaccharides, including cellulose, xyloglucan and pectin were further analyzed by heat map and some of them were verified by qRT-PCR analysis. **Figure 5A** shows the log_2_fold-change of the FPKM value in different Ca treatments versus the control. Two cellulose synthase-like (*Csl*) genes (*CslE6* and *CslC12*) were identified to be involved in biosynthesis of cellulose and xyloglucan, and their expression level was concentration-dependently depressed by Ca. Additionally, a xyloglucan galactosyltransferase gene (*GT11*) associated with xyloglucan biosynthesis was up-expressed under Ca supply. Xyloglucan endotransglucosylases/hydrolases (XTHs) are a class of enzymes with an extremely important role in the modification of cellulose-xyloglucan network (the skeleton of cell wall). Three *XTH* genes were identified to change significantly, with up-expression of *XTH22* and *XTH23* under Ca supply and maximal promotion at 0.5 mM Ca concentration, while down-expression of *XTH32* and maximal inhibition at 0.25 mM Ca concentration. These results implied great modification in the cellulose-xyloglucan skeleton under the regulation of related genes.

Among the selected cell wall genes, quite a few were associated with pectin metabolism. Specifically, two genes (galacturonosyltransferase-like 9 and 10, *GATL9* and *GATL10*) were identified to be involved in pectin biosynthesis and both of them were dose-dependently up-expressed by Ca. Meanwhile, under Ca supply, the expression was also increased for a polygalacturonase gene (*PG*) related to pectin degradation, but decreased for the gene inhibiting the activity of polygalacturonase (polygalacturonase inhibitor, *PGIP*). These results indicated that pectin metabolism, whether biosynthesis or degradation, is much more active in the presence of Ca. Moreover, we identified three pectin methylesterases (*PME18, PME41*, and *PMEU1*, which regulate pectin demethylation) and two pectin methylesterase inhibitors (*PMEI9* and *PMEI10*, which encode proteins restraining the activity of PMEs). All the *PMEs* and *PMEIs* were down-expressed under Ca treatment except for *PME41*, whose expression was much higher in the presence of Ca and increased with Ca concentration. The differential expression of these genes can lead to alterations in the pectin structure in different Ca treatments. The reliability of RNA-Seq data was verified by qRT-PCR analysis of the expression levels of *PME18, PME41, PMEU1, PMEI9, PMEI10* and *PG*, and the qRT-PCR results were consistent with RNA-seq results (**Figure 5B**).

### 3.6 Correlations of fluorine with calcium and cell wall components

**Figure 6:**
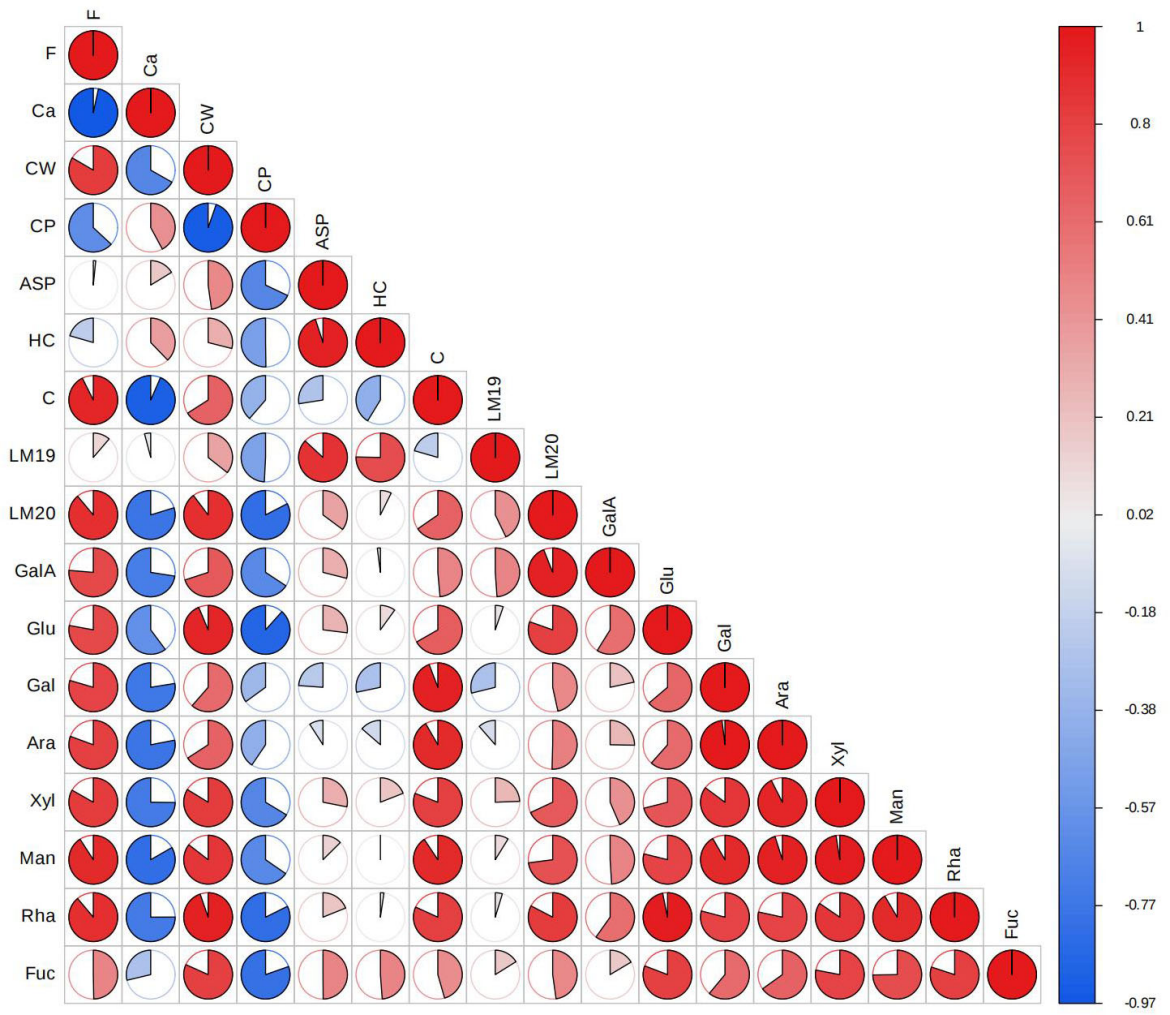
Heat map for Pearson correlation analysis of fluorine, calcium and cell wall fractions.

The relationship of F with Ca and cell wall components was investigated by Pearson correlation analysis of the ions and materials content. The results were presented with a heat map (**Figure 6**) and the Pearson correlation coefficient values were shown in **Supplementary Table S3**. The content of F and Ca in tea leaves showed a significant negative correlation with a correlation coefficient of -0.97 under different Ca concentrations. Moreover, the F was positively correlated with most of the cell wall materials, with a significant correlation with cellulose, mannose, rhamnose, and LM20-labled pectin. In order to further characterize the relationship between F and Ca and cell wall, we developed a multiple linear regression (MLR) model using F content as a dependent variable while Ca and cell wall materials as independent variables. Through MLR analysis, we obtained the formula: F=0.22GalA+0.26Fuc-0.73Ca-0.07LM19, demonstrating that Ca, fucose, galacturonic acid and LM19-labeled pectin play a more important role than other substances in F enrichment in tea plants, which deserve more attention in future studies. All the above results indicate that the variation of F accumulation was probably attributed to the changes in Ca and cell wall fractions in tea leaves under different Ca supply conditions.

## 4 Discussion

### 4.1 Foliar calcium application produces beneficial effect on the physiology of tea plants

Considering that the tea plant is sensitive to Ca (Liang et al., 2021), we assessed the physiological status of the tea plant by determining the MDA content and the SOD enzyme activity in tea leaves at the end of the treatment. The MDA is a marker of the lipid peroxidation and oxidative stress while the SOD is a key enzyme that helps plants resisting various stresses, both two are considered as important stress indicators (Akash et al., 2023). The results showed that the MDA content was decreased while the SOD activity was increased significantly with Ca concentration (**Figures 1A, B**), indicating that our current Ca treatments not only did not harm but also benefited the physiology of tea plants. Recently, some researchers also reported that the application of calcium in proper concentrations promoted the growth and physiological status of tea plants (Luo et al., 2019) and helped the tea plant resist stressful environments like cold and heat stresses (Chen et al., 2024; Malyukova et al., 2022). Therefore, it is of great practical significance to study the regulation mechanism of calcium on the growth, development, stress response and so on in tea plant.

### 4.2 Foliar calcium application can effectively reduce fluorine accumulation in tea plants

The characteristic of F hyper-accumulation in tea plant leaves has caused a safety concern for tea drinkers, suggesting the necessity to find some effective measures to reduce F content in tea leaves, such as reducing F absorption and accumulation in tea plant by applying exogenous substances (Cai et al., 2016). Many studies have shown that Ca can significantly reduce F content in tea plants through hydroponics or soil culture (Ruan et al., 2004; Luo et al., 2019). The current study examined the effect of foliar Ca spray at different concentrations on F accumulation in tea plant leaves, and foliar Ca application was shown to be effective to weaken F enrichment in tea plant (**Figure 1D**), agreeing with previous literature. Although the mechanism by which Ca lessens F in tea plant remains unclear, we speculate it should be different in varied calcium application methods. When calcium is applied to the roots of tea plants (e.g. hydroponics and soil culture), the reduction effect of Ca on tea F may attributed to the reduced root absorption induced by disturbed Ca signaling (Zhang et al., 2015). While in current study, the leaves are the target of Ca application, the Ca is probably to reduce the tea F by affecting the F storage capacity of tea leaves. The reasons underlying the conjecture can be stated as follows. The tea plant has a strong ability to absorb fluorine from the soil through its roots and readily transfer it to the leaves within a short uptake time (Ruan et al., 2003; Zhang et al., 2013), and the leaf organ is the main enrichment site of F accounting for 98% of the F in tea plant. Although the F in soil or nutrient solution has to be absorbed and transported by root and stem organs before reaching the leaf organ, the accumulation of F in tea plants is mainly attributed to the combination ability of F by tea leaves (Huang et al., 2016). Studies have shown that the cell wall is the main binding site of F (71%-92%) in tea leaves and the characteristics of cell wall composition and structure are the key factors that determine the F accumulation capacity of tea leaves (Luo et al., 2021). Ca is an important structural component of plant cell walls, which also plays an important role in regulating the cell wall structure and composition (Min et al., 2016; Hou et al., 2022). Studies have shown that foliar calcium spraying has a greater impact on the leaves cell wall structure (Wu et al., 2023). Our research found that the Ca content of tea leaves was significantly increased by foliar Ca spray of different concentrations (**Figure 1C**), indicating that the tea plant leaves can absorb and utilize Ca like roots. The foliar Ca can be absorbed by tea leaves through stomata, epidermal hydrophilic pores, and intercellular ligaments (Li et al., 2009). Thus we assume that the Ca entering the leaves may cause changes in the leaf cell wall structure and reduced the capacity of leaves to hold fluorine transported from the underground parts of the tea plant. Therefore, next we performed an in-depth analysis by revealing the relationship between F level and changes of cell wall in tea plant leaves under Ca regulation.

### 4.3 Fluorine enrichment in tea plant leaves is closely related to changes of tea leaf cell wall composition and structure under calcium regulation

Cellulose, hemicellulose and pectin are the three main types of polysaccharides to constitute the plant cell wall, with cellulose forming the skeleton structure in the form of microfibril, hemicellulose inlaid with the microfibril in the form of non-covalent bonds, and pectin filling the skeleton in a gel-like state (Albersheim et al., 2010). The results of the cell wall content and the composition of its polysaccharides and monosaccharides implied that foliar Ca spray may inhibit cell wall extension (**Table 1**; **Figure 2**), consistent with the previous studies in Nitella (Metraux and Taiz, 1977) and wheat (Zakir et al., 2005). As an important cell wall structure component, Ca participates in the gelation of pectin through cross-linking with low-methylated pectin chains, which is essential for immobilization of cellulose and hemicellulose to ensure cell wall stability, but also causes the stiffening of cell wall to impede its extension (Sarbjit and Robert, 1990; Blamey, 2003). The Ca cross-linked pectin was able to be extracted by Ca chelator as the chelate pectin fraction (Cybulska et al., 2015). In the present study, the chelate pectin level was found to be notably increased by exogenous Ca application, contrary to changes in total pectin content (**Table 1**). This result was consistent with the previous studies on Ca application to Jujube fruit (Cun et al., 2023) and herbaceous peony (Li et al., 2012), where Ca was found to increase the content of ion-bound pectin (chelate pectin) and inhibit the content of other pectin types. Based on above results and discussion, Ca can be speculated to inhibit the cell wall extension of tea plant by promoting the Ca cross-linked pectin structure. Correlation analysis revealed a strong positive correlation of F content in tea leaves with the contents of cell wall and its main component cellulose (**Figure 6**). Previous studies on F subcellular distribution have demonstrated that cell wall is the main part for F binding in tea plant leaves (Hu et al., 2019; Luo et al., 2021). Moreover, a positive correlation was observed between F and cell wall materials, especially cellulose and pectin in tea plant treated with different F concentrations (Luo et al., 2021). In the current study, these findings were further confirmed, suggesting that the dampening effect of Ca on F may be related to the lessened cell wall materials. The simultaneous fluctuation of cell wall polysaccharides and F content suggests that the tea leaf cell wall has a strong capacity to absorb and adjust F.

Methylesterification, the most important structural feature of pectin, has a close relationship to Ca ions (Wolf et al., 2009). Homogalacturonan (HG) is a major component of pectin, which is synthesized in a highly methylesterified form and can be de-esterified by PMEs to form low-methylesterified structures capable of cross-linking Ca. In the current study, the PME activity was increased and both the immunostaining semi-quantitative high-methylesterified pectin and DM value were lessened by Ca spray (**Figures 3**, **4**), revealing that foliar Ca application can promote de-methylesterification of tea leaf cell wall by activating the PME enzyme, which may be attributed to the requirement to provide more binding sites for superfluous Ca in the cell wall of tea plant leaves. Consistent with our findings, Li (2021) found that Ca treatment could activate PME activity and reduce the DM value, promoting pectin cross-linking with Ca and structure remodeling.

Based on MLR analysis, key fractions influencing F accumulation are selected, including the content of Ca, fucose, galacturonic acid and low-methylesterified pectin. It is worth noting that all the four fractions are important structural components of pectin (Cao et al., 2020), reflecting the pectin should play a pivotal role in reducing F among cell wall polysaccharides. The pectin mainly consists of the unbranched homopolymer chain (HG) of galacturonic acid residues linked by (1→4) glycosidic bond and a small number of branched structures constructed with a greater diversity of mono-sugars, such as rhamnose, arabinose, and galactose and fucose, thereinto the adjacent low-methylesterified pectins are able to be connected by Ca by binding to free carboxyl groups of galacturonic acid (Min et al., 2016). Pectin was shown to be the principal structure for F binding in the tea plant cell wall (Luo et al., 2021). Moreover, a positive association was observed between the HG type pectin amount and F content in the tea plant leaves treated with different exogenous F concentrations (Luo et al., 2020). In the present study, both the content of galacturonic acid (basic unit of HG) and F levels were shown to be decreased by exogenous Ca application (**Figures 1B**, **2**), providing further evidence for the tight association between HG and F levels. Beyond the content, the methylesterification structure of pectin was another influential factor of F accumulation. In the study by Luo et al. (2021), F treatment was found to promote the formation of low-methylesterified pectin and inhibit the formation of high-methlyesterified pectin, inferring that F probably stays chelated in the low-methylesterified pectin structure. However, in the present study, the DM value was observed to decrease with increasing Ca concentration, indicating that enhanced pectin de-methylesterification might weaken F accumulation in tea plant leaves. Hence, the effect of pectin methylesterification process on F binding may be more complex. One possible explanation is that pectin demethylesterification can produce rich de-esterified structures due to the varied distribution pattern of demethylgalacturonic acid residues in HG, leading to more intricate and superior pectin structure changes and weakening F binding (Celus et al., 2017). Another more plausible explanation is derived from the increased Ca cross-linked chelate pectin, which requires pectin de-methylesterification to form its structure, and its increase may enable tighter intercellular adhesion and lower cell wall porosity, thus less space for F binding in the tea plant cell wall (Liu et al., 2022).

### 4.4 Foliar calcium application enables large fluctuations in expression of genes involved in cell wall metabolism

As a vital element for plant growth and development, the change of Ca concentration in the external environment can greatly affect the gene expression and consequently the metabolism and physiological processes in plants (Hu et al., 2023). In current study, foliar Ca application was shown to have great influences on the expression of genes associated with cell wall metabolism (**Figure 5**), which supports our findings that Ca spraying caused strong changes in cell wall structure and materials in tea plant. Ca may induce changes in the structure and components of tea plant cell wall by regulating the expression of cell wall-related genes. For example, the down-expression of two cellulose synthase superfamily genes (*CslE6* and *CslC12*) echoed the content decrease of cellulose in tea plant leaves under varying Ca doses. The tea plant cell wall is structured by a cellulose-hemicellulose skeleton infilled with pectin materials (Somerville et al., 2004). According to available literature, many studies have been performed on the effect of Ca on the expression of pectin-related genes, while few reports are available about the influence of Ca on cellulose- or hemicellulose- related genes. In a recent study, Ca was reported to regulate a cellulose synthase-like gene (*CSLD3*) at the post-translational level in apple fruit (Xu et al., 2022). Here, six genes (*CslE6*, *CslC12*, *GT11*, *XTH23*, *XTH22*, and *XTH32*) in tea plant were identified to be involved in the regulation of cellulose-hemicellulose network induced by Ca, enriching the information for further understanding of Ca influence on the skeleton structure of plant cell wall. The influence of Ca spraying on pectin-related genes expression was investigated in fruity plants such as cherry (Wu et al., 2023), apple (Xu et al., 2022), citrus (Huai et al., 2022), and grape (Martins et al., 2020). All of these studies reported that Ca could suppress the expression of genes associated with pectin degradation (*PGs* and *PMEs*), resulting in increased cell wall integrity and fruit firmness. However, our results showed a more complex picture in tea plant leaves, i.e., exogenous Ca application could simultaneously exert up-regulation effects on genes related to pectin biosynthesis (*GATL9*, *GATL10*) and pectin degradation (*PG* and *PME41*), which reflected a difference in species and tissues. Pectin is the most structurally complex and flexible and functionally abundant constituent in plant cell wall (Magdalena, 2011). Our results suggested that Ca application has a greater impact on pectin structure reorganization than its content in tea plant leaves.

## 5 Conclusion

**Figure 7:**
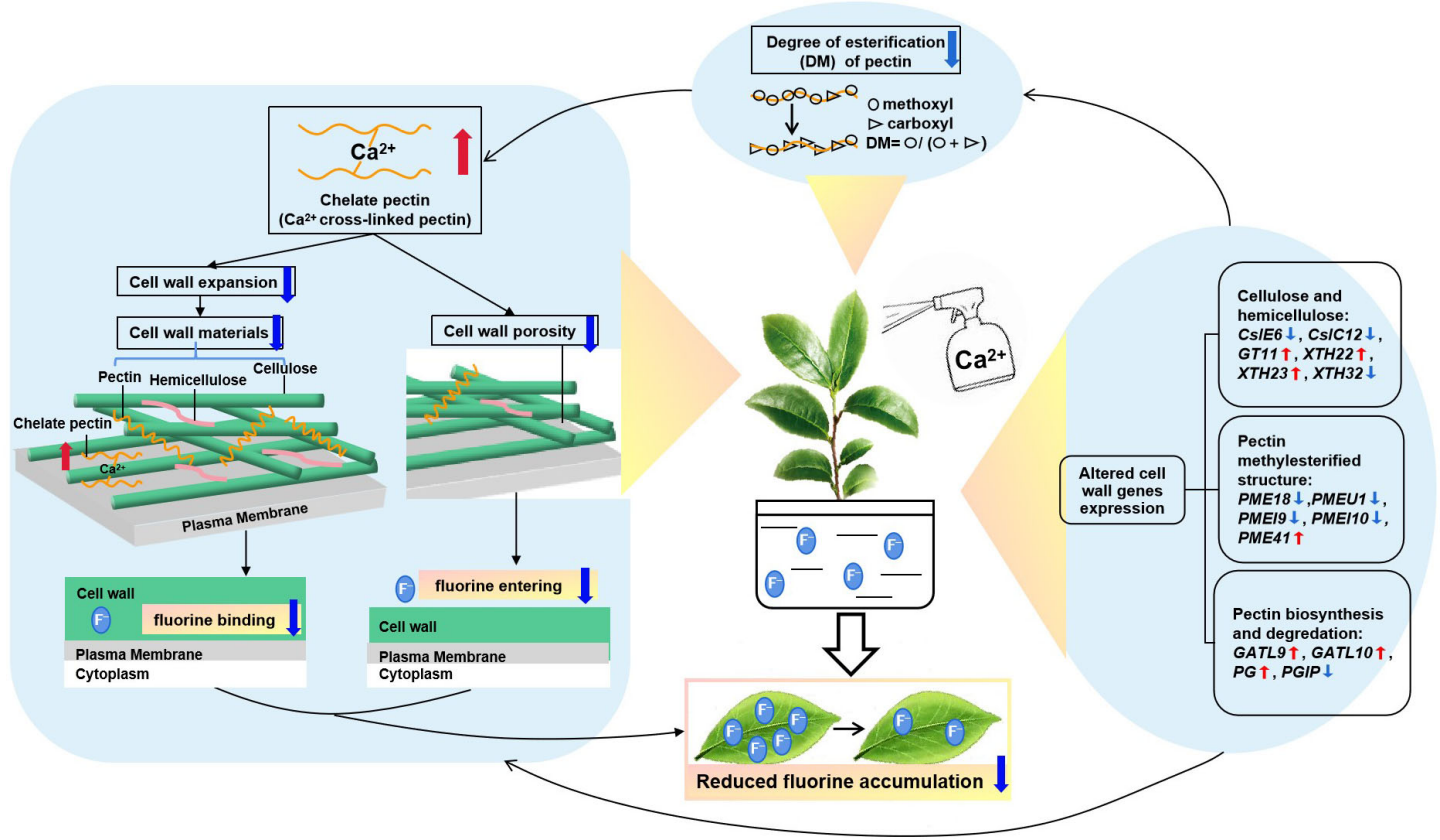
A putative model of foliar calcium application reducing the fluorine content in tea plant. The calcium-induced fluctuations of cell wall-related genes could influence the reorganization of wall polysaccharides and alter the methylesterification structure, causing morphological changes to reduce cell wall extension and porosity, thereby lessening fluorine binding in tea plant leaves.

In conclusion, foliar Ca spray can decrease F accumulation in tea plant leaves by changing cell wall composition, structure and related-gene expression, i.e., Ca had an overall suppressing effect on the amount of cell wall polysaccharides and monosaccharides, thereby lessening F binding by the tea plant cell wall. Additionally, Ca can decrease the DM value of pectin methylesterification structure, leading to an increase of chelate pectin accumulation, further restricting cell wall extension and lessening porosity, thereby reducing the entry of F into the cell wall or the cellular interior. The above two factors are the possible contributors to the defluorination effect of Ca in tea plants. This process was shown to be involved in by several identified key genes related to the biosynthesis, degradation and reorganization of cell wall polysaccharides, which deserves to be more deeply explored in further studies. A model of foliar Ca spraying for reducing F content in tea plant leaves was constructed in **Figure 7**. This study provides novel insights into the mechanisms by which Ca reduces F accumulation in tea plant leaves and has guiding significance for finding effective measures to reducing F content in tea plant.

## Data Availability

The datasets generated and analyzed in the current study are available in the NCBI repository, accession number: PRJNA1065635, https://www.ncbi.nlm.nih.gov/sra/PRJNA1065635.
