# Supplementary material for: Foliar calcium application reduces fluorine accumulation in tea plant by regulating cell wall structure and gene expression
Source: Front Plant Sci. 2025 Jan 3;15:1443439. doi: 10.3389/fpls.2024.1443439 (PMC11739158; doi:10.3389/fpls.2024.1443439)
Supplement: Supplementary file 1 [file DataSheet1.docx]

Supplementary Material


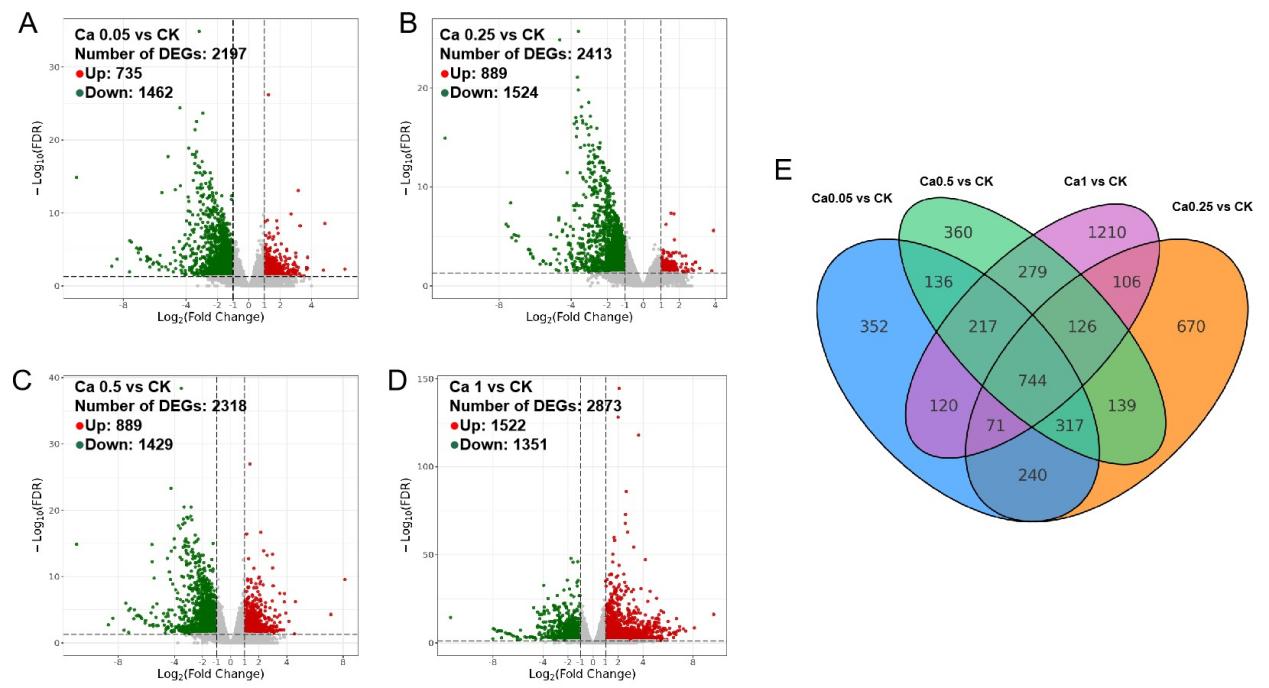


**Supplementary Figure 1**.Volcano plot for the number of DEGs of Ca0.05 (a), Ca0.25 (b), Ca0.5 (c), Ca1 (d) versus the CK. Figure S1E shows the Venn diagram for comparison groups of different calcium treatments versus the CK, respectively.


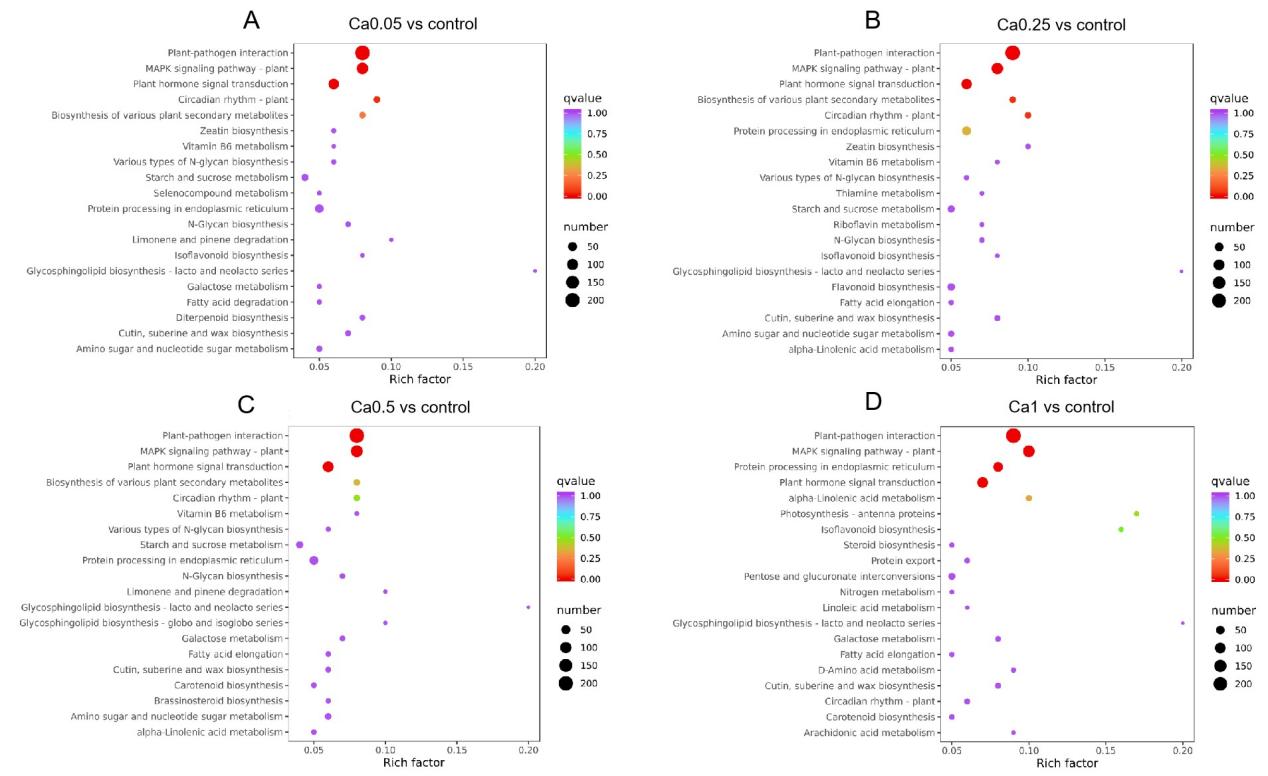


**Supplementary Figure 2**. The top 20 significantly enriched KEGG pathways in the comparison of Ca0.05 (a), Ca0.25(b), Ca0.5(c) and Ca1(d) versus the control, respectively.

**Supplementary Table 1**. Primer sequences for qRT-PCR

| Gene | Forward | Reverse |
| --- | --- | --- |
| PME18 | TTGGCATCGTTGAGGGTCT | CAGTGGGAACACGGAAAGC |
| PME41 | CCTCCCACCACAGTCCTTA | GATGACCCAAATGGCAAAG |
| PMEU1 | GTTATTGGTGGCGTTTCGG | TCATCTGATGCCTTGGAGG |
| PMEI9 | CGGAACACTGTATTCTTTG | TCTTTGGAGCATCATAACC |
| PME10 | GGATGAACTACACGCTCTG | ATTGATGGACTGATAGGGA |
| PG | CCACCAATCAGAATAGACCC | GAGAACGAGACGCAACACC |

**Supplementary Table 2**. Statistics on the data quality of transcriptome sequencing

| **Sample** | **Raw Reads** | **Clean Reads** | **Clean Base(G)** | **Error Rate (%)** | **Q20 (%)** | **Q30 (%)** | **GC Content (%)** |
| --- | --- | --- | --- | --- | --- | --- | --- |
| CK_1 | 46294552 | 42295652 | 6.34 | 0.03 | 97.56 | 93.27 | 43.88 |
| CK_2 | 219887160 | 208566988 | 31.29 | 0.03 | 97.42 | 92.99 | 43.5 |
| CK_3 | 49589252 | 47164776 | 7.07 | 0.03 | 97.24 | 92.58 | 43.32 |
| Ca1_1 | 42665962 | 40636780 | 6.1 | 0.03 | 97.29 | 92.69 | 43.37 |
| Ca1_2 | 48857430 | 46421982 | 6.96 | 0.03 | 97.49 | 93.06 | 43.71 |
| Ca1_3 | 44256646 | 42311498 | 6.35 | 0.03 | 97.28 | 92.68 | 43.64 |
| Ca2_1 | 42572388 | 40932736 | 6.14 | 0.03 | 97.22 | 92.55 | 43.38 |
| Ca2_2 | 45720992 | 43307468 | 6.5 | 0.03 | 97.45 | 93.03 | 43.65 |
| Ca2_3 | 56887358 | 53359252 | 8 | 0.03 | 97.61 | 93.42 | 43.66 |
| Ca3_1 | 48322202 | 45414408 | 6.81 | 0.03 | 97.29 | 92.72 | 43.49 |
| Ca3_2 | 42856632 | 40234156 | 6.04 | 0.03 | 97.3 | 92.71 | 43.73 |
| Ca3_3 | 45781900 | 43228316 | 6.48 | 0.03 | 97.64 | 93.47 | 43.67 |
| Ca4_1 | 61792504 | 58770778 | 8.82 | 0.03 | 97.16 | 92.37 | 43.71 |
| Ca4_2 | 62217750 | 58722980 | 8.81 | 0.03 | 97.58 | 93.24 | 43.68 |
| Ca4_3 | 65995584 | 61749732 | 9.26 | 0.03 | 97.45 | 93.05 | 43.7 |

Note: Raw reads, the number of raw data reads; Clean Reads, the number of high-quality reads filtered from the original data; Clean Bases, the total number of bases of high quality reads; Error Rate, the overall sequencing error rate; Q20, the percentage of bases with Qphred value at least 20 in the total number of bases; Q30, the percentage of bases with Qphred value at least 30 in the total number of bases; GC Content, the percentage of the sum of the quantities of G and C in the total number of bases in high quality reads.

**Supplementary Table 3**. Pearson correlation coefficient values among fluorine, calcium and cell wall components.

|  | F | Ca | CW | CP | ASP | HC | C | LM19 | LM20 | GalA | Glu | Gal | Ara | Xyl | Man | Rha | Fuc |
| --- | --- | --- | --- | --- | --- | --- | --- | --- | --- | --- | --- | --- | --- | --- | --- | --- | --- |
| F | 1.00 | -0.97 | 0.83 | -0.63 | 0.02 | -0.21 | 0.93 | 0.11 | 0.89 | 0.76 | 0.78 | 0.80 | 0.81 | 0.83 | 0.91 | 0.89 | 0.50 |
| Ca | -0.97 | 1.00 | -0.67 | 0.42 | 0.16 | 0.38 | -0.93 | -0.04 | -0.80 | -0.73 | -0.60 | -0.78 | -0.78 | -0.75 | -0.83 | -0.75 | -0.29 |
| CW | 0.83 | -0.67 | 1.00 | -0.95 | 0.48 | 0.29 | 0.66 | 0.36 | 0.90 | 0.70 | 0.94 | 0.61 | 0.66 | 0.84 | 0.85 | 0.95 | 0.82 |
| CP | -0.63 | -0.42 | -0.95 | 1.00 | -0.68 | -0.50 | -0.39 | -0.49 | -0.83 | -0.66 | -0.88 | -0.35 | -0.41 | -0.66 | -0.65 | -0.82 | -0.80 |
| ASP | 0.02 | 0.16 | 0.48 | -0.68 | 1.00 | 0.95 | -0.27 | 0.87 | 0.35 | 0.29 | 0.27 | -0.24 | -0.09 | 0.28 | 0.13 | 0.19 | 0.50 |
| HC | -0.21 | 0.38 | 0.29 | -0.50 | 0.95 | 1.00 | -0.41 | 0.75 | 0.07 | -0.02 | 0.10 | -0.28 | -0.14 | 0.19 | 0.01 | 0.02 | 0.49 |
| C | 0.93 | -0.93 | 0.66 | -0.39 | -0.27 | -0.41 | 1.00 | -0.21 | 0.65 | 0.49 | 0.67 | 0.94 | 0.92 | 0.81 | 0.91 | 0.82 | 0.46 |
| LM19 | 0.11 | -0.04 | 0.36 | -0.49 | 0.87 | 0.75 | -0.21 | 1.00 | 0.43 | 0.49 | 0.05 | -0.29 | -0.11 | 0.24 | 0.09 | 0.05 | 0.16 |
| LM20 | 0.89 | -0.80 | 0.90 | -0.83 | 0.35 | 0.07 | 0.65 | 0.43 | 1.00 | 0.94 | 0.80 | 0.46 | 0.50 | 0.68 | 0.73 | 0.83 | 0.48 |
| GalA | 0.76 | -0.73 | 0.70 | -0.66 | 0.29 | -0.02 | 0.49 | 0.49 | 0.94 | 1.00 | 0.59 | 0.22 | 0.25 | 0.44 | 0.49 | 0.60 | 0.16 |
| Glu | 0.78 | -0.60 | 0.94 | -0.88 | 0.27 | 0.10 | 0.67 | 0.05 | 0.80 | 0.59 | 1.00 | 0.64 | 0.62 | 0.71 | 0.79 | 0.97 | 0.81 |
| Gal | 0.80 | -0.78 | 0.61 | -0.35 | -0.24 | -0.28 | 0.94 | -0.29 | 0.46 | 0.22 | 0.64 | 1.00 | 0.98 | 0.85 | 0.92 | 0.79 | 0.61 |
| Ara | 0.81 | -0.78 | 0.66 | -0.41 | -0.09 | -0.14 | 0.92 | -0.11 | 0.50 | 0.25 | 0.62 | 0.98 | 1.00 | 0.93 | 0.95 | 0.78 | 0.65 |
| Xyl | 0.83 | -0.75 | 0.84 | -0.66 | 0.28 | 0.19 | 0.81 | 0.24 | 0.68 | 0.44 | 0.71 | 0.85 | 0.93 | 1.00 | 0.98 | 0.85 | 0.78 |
| Man | 0.91 | -0.83 | 0.85 | -0.65 | 0.13 | 0.01 | 0.91 | 0.09 | 0.73 | 0.49 | 0.79 | 0.92 | 0.95 | 0.98 | 1.00 | 0.92 | 0.75 |
| Rha | 0.89 | -0.75 | 0.95 | -0.82 | 0.19 | 0.02 | 0.82 | 0.05 | 0.83 | 0.60 | 0.97 | 0.79 | 0.78 | 0.85 | 0.92 | 1.00 | 0.80 |
| Fuc | 0.50 | -0.29 | 0.82 | -0.80 | 0.50 | 0.49 | 0.46 | 0.16 | 0.48 | 0.16 | 0.81 | 0.61 | 0.65 | 0.78 | 0.75 | 0.80 | 1.00 |

Note: The red numbers indicate a significant correlation at the 0.05 level.
